# Long-term safety of childhood growth hormone treatment: evidences from real-world study and future directions

**DOI:** 10.1007/s12519-024-00862-7

**Published:** 2024-12-12

**Authors:** Cai Zhang, Yan Liang, Xiao-Ping Luo

**Affiliations:** 1 Department of Pediatrics, Tongji Hospital, Tongji Medical College, Huazhong University of Science and Technology, Wuhan, 430030 People’s Republic of China; 2 Hubei Key Laboratory of Pediatric Genetic Metabolic and Endocrine Rare Diseases, Wuhan, China

## Background

Recombinant human GH (rhGH) was first approved to treat children with growth hormone deficiency (GHD) by Food and Drug Administration (FDA) in the United States in 1985. The indications subsequently expanded to growth failure secondary to chronic renal failure, Turner syndrome, Prader‒Willi syndrome, small for gestational age (SGA) without catch-up growth, idiopathic short stature (ISS), SHOX deficiency, Noonan syndrome and so on [1]. The efficacy and the safety of rhGH have been proven in randomized controlled trials (RCTs), which are considered the highest level of evidences in clinical practice. However, due to the strict criteria and short observational time, RCTs could not fully reflect the reality of rhGH, especially in terms of safety [2].

Real-world evidences (RWEs) have become increasingly important in clinical practice. They are based on observational data from the daily medical world, representing the real situation in clinical practice [3]. RWEs are regarded to be complementary to RCTs. For example, RCTs have advantages in evaluating the efficacy of a drug, while RWEs can focus on the epidemiology, effectiveness, safety, or costs of treatments [4]. Since rhGH has been used in short children for almost 4 decades, numerous clinical information has been collected from hospital-based clinical systems, insurance company records, as well as post-marketing databases established and managed by pharmaceutical industries.

There are several large long-term real-world databases in the world. The Kabi/Pfizer International Growth Database (KIGS), which was established in 1987, was a large international observational database to monitor the long-term clinical outcomes and safety in children treated with Genotropin® (Pfizer, USA) [5]. The NordiNet International Outcome Study (ISO) and American Norditropin Studies: Web-Enabled Research (ANSWER) Program were non-interventional, multicenter registry studies monitoring the long-term outcomes of Norditropin® (NovoNordisk, Denmark). The NordiNet IOS was ongoing from 2006 to 2016 across 22 European and Middle Eastern countries, while the ANSWER Program operated from 2002 to 2016 in the United States [6]. The Genetics and Neuroendocrinology of Short Stature International Study (GeNeSIS) sponsored by Eli Lilly and Company (USA), was conducted in 30 countries between 1999 and 2015. It included not only patients treated with rhGH, but also a small portion of untreated patients [7]. Moreover, the large cross-European cohort, the Safety and Appropriateness of Growth Hormone Treatments in Europe (SAGhE) established in 2009, conducted the study of rhGH-treated patients with follow-up and analysis independent from the pharmaceutical industry [8].

Over the past few years, there have been an upsurge of publications based on large long-term observational studies focusing on the safety of childhood rhGH treatment. We aimed to summarize the RWEs of rhGH treatment in children and discuss possible directions in the future.

## Cancer risk

The correlation between growth hormone (GH) excess and cancer is controversial. Evidence from patients with acromegaly has suggested a relationship between increased circulating GH levels and elevated risks of colorectal and thyroid cancer [9]. Moreover, the effector of rhGH, insulin-like growth factor 1 (IGF-1), was proved to be positively associated with increased risks of different cancers, such as thyroid, colorectal, breast and prostate cancer [10]. Thus, rhGH treatment has led to concerns regarding increased risks of cancers.

The potential link between rhGH therapy and an increased risk of cancer in children was first reported in Japan in the 1980s that described the development of leukemia in children treated with GH. However, at least half of these patients had predisposing risk factors for leukemia, and the risk of leukemia was not increased in children without risk factors. In 2002, a study of 1,848 patients treated with GH during childhood and early adulthood reported increased risks of colorectal cancer and Hodgkin lymphoma, but the absolute number of cases was extremely low.

Since then, large observational real-world studies have been conducted to investigate the risk of cancer in rhGH-treated children. Most of the studies reported no increased risk of cancer in patients without underlying risk factors [5, 11]. The Safety and Appropriateness of Growth Hormone Treatments in Europe (SAGhE) cohort recruited 23,984 patients treated with rhGH in childhood were recruited for different reasons. The final results were published in 2017. The average time of follow-up for cancer incidence was 14.8 years per patient, and the mean age at the end of follow-up was 25.8 years. The results revealed that the risk of cancer was significantly increased in patients with previously diagnosed cancer. The standardized incidence ratio (SIR) was 1.4 with a 95% confidence interval (CI) ranging from 1.1 to 1.9. The overall risk of cancer was not increased in patients with growth failure only. However, for patients with other diseases, such as chronic renal diseases and Turner syndrome, the risks of bone and bladder cancer were significantly increased. Additionally, cancer risk was unrelated to the duration or cumulative dose of rhGH treatment, but for patients with previous cancer, the mortality risk of cancer increased significantly with increased doses of rhGH [12].

Most recently, a study from Sweden investigated the risk of neoplastic events of rhGH treatment during childhood in more than 3400 patients with GHD, SGA and ISS compared to healthy controls. The patients were followed for up to 35 years, with a median follow-up time of 19.8 years. The results showed that in early to mid-adulthood, a moderately increased risk for neoplastic events was observed in patients treated with rhGH, but no elevated risk of malignant neoplastic events was found. Additionally, a longer treatment duration, but not the mean or cumulative dose, was associated with an increased risk of neoplastic events [13].

Thus far, according to the available reports, rhGH treatment in childhood does not appear to increase the risk of cancer in early adulthood for patients with low risk. Nevertheless, more high-quality studies with long observational times are needed to confirm these conclusions. More importantly, there is a shortage of high-quality evidence regarding the cancer risk associated with rhGH treatment in patients who have predisposing factors for cancer.

## Cardiovascular risk

The GH/IGF-1 axis plays an important role in the cardiovascular system. Both GHD and excessive GH secretion can lead to an increased risk of cardiovascular diseases [14]. The underlying mechanisms involve direct effects of GH on endothelial function, as well as the indirect regulation of lipid and glucose metabolism [15].

The concern regarding the cardiovascular risk associated with pediatric treatment with rhGH was first raised in 2012 by the French subgroup of SAGhE. In the French subgroup, a total of 6928 children with GHD, ISS, neurosecretory dysfunction and SGA were recruited. The mean follow-up time was 17.3 years. The results revealed a significant increase in mortality due to diseases of the circulatory system or subarachnoid or intracerebral hemorrhage in patients treated with rhGH compared with the general French population [16, 17]. However, the subgroups of Belgium, Netherlands and Sweden from the same SAGhE cohorts did not observe the increased mortality from cardiovascular or cerebrovascular diseases [18].

An observational study from Sweden investigated the association of childhood GH treatment with long-term cardiovascular diseases and the result was published in 2020. A total of 3408 patients with GHD, SGA, or ISS and 50,036 healthy controls were followed up, with a median follow-up time of 14.9 years. It showed that rhGH treatment during childhood in patients with GHD, SGA, or ISS was associated with increased risks of cardiovascular events in early adulthood, particularly in women. The hazard ratio (HR) for such events was 1.69 (95% CI 1.30-2.19), and the risk was particularly high in females with an HR of 2.05 (95% CI 1.31-3.20). Additionally, longer duration of rhGH treatment and total cumulative dose were related with higher risks for overall cardiovascular diseases [19].

To analyze the effect of childhood rhGH treatment on cardiovascular risk in SGA patients, a study from Netherlands longitudinally investigated the metabolic and cardiovascular health markers of adults born SGA during the 12 years of follow-up after the cessation of rhGH treatment. Comparison groups included adults born SGA with persistent short stature (SGA-S), adults born SGA and previously treated with growth hormone (SGA-GH), adults born SGA with spontaneous catch-up growth (SGA-CU) and adults born appropriate for gestational age (AGA). The results revealed that at approximately 30 years of age, SGA-GH adults had metabolic and cardiovascular health profiles similar to those of untreated adults born SGA or AGA. However, the sample sizes were small for the groups, as 167 SGA-GH patients, 50 SGA-S patients, 77 SGA-CU patients and 92 AGA adults were recruited for the study [20].

Thus, the effect of rhGH treatment in childhood on long-term cardiovascular risk is not clear. The inconsistency of the conclusions could be related to the heterogeneity of the studies, as well as the complexity of the confounders. Although the patients were followed up for more than 10 years, they were still young for the onset of cardiovascular disease. Noteworthy, the risk of cardiovascular disease varies among patients with different causes of short stature. For example, GHD patients without treatments have an increased risk of cardiovascular disease, and rhGH treatment was believed to improve cardiovascular risk in GHD patients [15]. Moreover, patients with SGA, Turner syndrome or Noonan syndrome have a greater risk of cardiovascular diseases [20–22]. The risk in patients with different causes should be assessed separately, and appropriate untreated groups should be recruited for comparison.

## Diabetes risk

GH inhibits the uptake and oxidation of glucose, increases gluconeogenesis and reduces insulin sensitivity. It is acknowledged as a key counter-regulatory hormone to insulin, and the propensity of GH to induce diabetes is further substantiated by the high prevalence of diabetes in patients with acromegaly. However, the impact of rhGH administration on overall glucose homeostasis is complex, partly due to the glucose-lowering influence of IGF-1[23].

In 2000, the Pharmacia and Upjohn International Growth Study (KIGS) study suggested a sixfold increase in the incidence of type 2 diabetes among children treated with rhGH, whereas the incidence of type 1 diabetes did not increase. Furthermore, the alteration in glucose metabolism persisted even after the discontinuation of rhGH [24]. However, the French SAGhE study, revealed no difference in the prevalence of diabetes between rhGH-treated patients with GHD, ISS, or SGA and the general population [25].

A report based on Genetics and Neuroendocrinology of Short Stature International Study (GeNeSIS), an open-label, multinational, observational, postauthorization safety study sponsored by Eli Lilly and Co. (Indianapolis, IN), was established in 1999. A report based on the Genetics and Neuroendocrinology of Short Stature International Study (GeNeSIS) analyzed the incidence of diabetes in rhGH-treated children. It included children treated with rhGH due to GHD, ISS, Turner syndrome, SGA, Prader–Willi syndrome, chronic renal insufficiency and so on. A total of 21,448 patients were included in the study, with a median follow-up time of 5.0 years. The results showed that the incidence of type 2 diabetes was higher in rhGH-treated children than that in the general population (SIR 3.77; 95% CI 2.24-5.96). Among the 18 patients who developed type 2 diabetes, 13 patients exhibited risk factors, including organic GHD, Turner syndrome, Prader–Willi syndrome, obesity, and preexisting insulin resistance or impaired glucose tolerance [7].

In general, rhGH treatment appears to be associated with an increased risk of type 2 diabetes in children with susceptible factors. However, owing to the absence of the untreated control group, the precise effects of rhGH on glucose metabolism need to be further investigated.

## Mortality risk

In 2012, the an analysis of the French subgroup of SAGhE revealed increased all-cause mortality, as well as mortality due to bone tumors and diseases of the circulatory system or subarachnoid or intracerebral hemorrhage [16]. However, the findings from the subgroup of Belgium, Netherlands and Sweden from the same SAGhE cohorts did not support these findings [18].

The full analysis of the entire dataset of the SAGhE cohort was reported in 2020. The cohort included 24,232 patients who were treated with rhGH during childhood, with up to 25 years of follow-up. The results indicated that all-cause mortality was not significantly increased in low-risk patients with GHD or ISS. In patients at moderate or high risk, such as those with clinically defined syndromes or pre-existing tumors, mortality was increased compared with in the general population. The standardized mortality ratio (SMR) was 3.8 (95% CI 3.3-4.4) and 17.1 (95% CI 15.6-18.7) for patients with moderate or high risk respectively. Additionally, mortality was not associated with the mean daily or cumulative dose of rhGH. This finding suggested that the increased mortality in patients with risk factors may not be related to rhGH treatment but may be due to the underlying diseases [26].

A study published in 2021 collected data from the NordiNet International Outcome Study (2006–2016, Europe) and the ANSWER Program (2002–2016, USA). In total, 37 702 patients were included in the study, with up to 10 years of follow-up. The patients were classified into 3 mortality risk groups on the basis of primary diagnosis. As reported, no increased risk of mortality was found in any risk group [6].

Overall, rhGH treatment does not seem to increase mortality risk in patients at low risk, but continued long-term surveillance is still needed to confirm this conclusion. Moreover, the mortality risk in patients with underlying disease should be further assessed.

## Future directions

Despite the growing number of real-world studies on the long-term safety of childhood rhGH treatment, there is notable heterogeneity in the methodological rigor and quality of these studies. Owing to the nature of long-term observational studies, issues such as the heterogeneity of the study populations, the presence of confounding factors, the integrity of the data and attrition rates can undermine the robustness and validity of the study outcomes. Therefore, the implementation of rigorous inclusion and exclusion criteria, standardized data collection procedures, the mitigation of confounding factors, and an adequately sized sample are instrumental in enhancing the quality of the research. Furthermore, the complexity of the data necessitates the involvement of professional statisticians and epidemiologists to guarantee the application of robust methodology, as well as accurate analysis and interpretation.

Regarding the long-term safety of rhGH treatment, the durations of follow-up in the studies were still short, particularly with respect to cancer and cardiovascular risks. Owing to the exceptionally low incidence of events, such as cancer and death, the outcomes of the statistical analyses lack reliability. Thus, extended follow-up times and expanded sample sizes are essential for achieving robust RWE in subsequent research endeavors. Moreover, patients with different etiologies of short stature, accompanying diseases and predisposing factors are subject to varying levels of risk for clinical conditions, such as cancer, cardiovascular disease and diabetes. In this circumstance, an untreated control group would be preferable for comparison over a healthy control group. Information on confounding factors, including familial history, genetic background, environmental exposure, and lifestyle, should be meticulously collected and analyzed. Additionally, the long-term safety of childhood rhGH therapy related to the dosage and duration of treatment needs to be further investigated. In patients with rare diseases, such as RASopathies, chromosomal breakage syndromes or DNA repair disorders, the long-term safety of rhGH therapy should also be exhaustively explored.

In conclusion, the long-term safety of childhood rhGH treatment seems to be established in patients at low risk, but long-term real-world studies with large sample sizes are essential for comprehensive validation. Furthermore, safety in patients with varying clinical conditions and confounding factors requires additional investigation.

## Data Availability

No datasets were generated or analyzed during the current study.
